# Cytomegalovirus Viremia in Renal Transplant Recipients After Influenza Vaccination

**DOI:** 10.7759/cureus.9680

**Published:** 2020-08-12

**Authors:** Akshay M Khatri, Ilan Berlinrut, Robin Koshy, Madhu Bhaskaran

**Affiliations:** 1 Infectious Diseases, Northwell Health, Manhasset, USA; 2 Nephrology and Transplant Nephrology, Northwell Health, Manhasset, USA

**Keywords:** cytomegalovirus (cmv), influenza vaccine, renal transplant, viremia

## Abstract

Vaccination with the inactivated influenza vaccine is routinely recommended for all patients before and after transplant, with reduction in complications noted in transplant recipients. The vaccine is relatively well tolerated with few mild side effects. Cytomegalovirus (CMV) infection can reactivate in both solid organ transplant and hematopoietic stem cell transplant recipients, with some patients progressing to disease. There are multiple factors known to contribute to reactivation and subsequent CMV disease, however vaccination has not been reported as a specific risk factor.

We report on two renal transplant recipients who were seen to develop CMV viremia and CMV disease after receiving the Influenza vaccine. We review the literature regarding viremia occurring after vaccination in HIV patients (a similar group of immunocompromised patients).

## Introduction

The inactivated Influenza vaccine is recommended in all solid organ transplant recipients (SOTRs) and it can be administered either before or one month after transplantation [1]. In SOTRs, it was associated with a decrease in severity of influenza disease (as determined by occurrence of pneumonia and incidence of intensive care unit admission). The vaccine is relatively well tolerated - there have been reports of mild reactions, with some vaccine strains being associated with risk of rejection [1].

Cytomegalovirus (CMV) can reactivate in both solid organ transplant and hematopoietic stem cell transplant recipients [2, 3]. There are multiple patient- and therapy-related factors known to contribute to reactivation and subsequent CMV disease [2-6].

Vaccination has reported to cause viremia with human immunodeficiency virus (HIV) in patients who are HIV-infected, even those who are on potent anti-retroviral therapy (ART) [7, 8]. Despite the extensive research, vaccination has not been reported to be a risk factor for CMV viremia in transplant recipients.

In this case report, we describe two cases of kidney transplant recipients who received the influenza vaccine and developed cytomegalovirus (CMV) viremia thereafter. Due to the paucity of literature regarding viremia after vaccination in transplant recipients, we review the studies reporting viremia occurring after vaccination in HIV patients (a similar group of immunocompromised patients).

## Case presentation

Patient 1

A 50-year-old female with past medical history of hypertension, autosomal dominant polycystic kidney disease and end-stage renal disease (on hemodialysis) underwent a renal transplant from a living unrelated donor in June 2016.

She received the influenza vaccine in November 2017 at the outpatient transplant nephrology clinic. She presented to her next appointment eight days later with complaints of fevers with chills, night sweats, lethargy, headaches, myalgias, nausea, anorexia, left-sided pleuritic chest pain and multiple episodes of watery, non-bloody diarrhea. She was directed to the emergency department for further evaluation.

She denied sick contacts, recent travel, medication changes or medication non-compliance. At the time of admission, she was on prednisone 5 mg PO (oral) QD, mycophenolate mofetil (MMF) 360 mg PO QID, tacrolimus 0.5 mg PO BID.

Her vitals on admission were significant for temperature 101.7 F, heart rate 100/minute, blood pressure 118/79 mm Hg, respirations 18/minute and oxygen saturation 98% on room air. Physical exam revealed no acute abnormalities.

**Figure 1:**
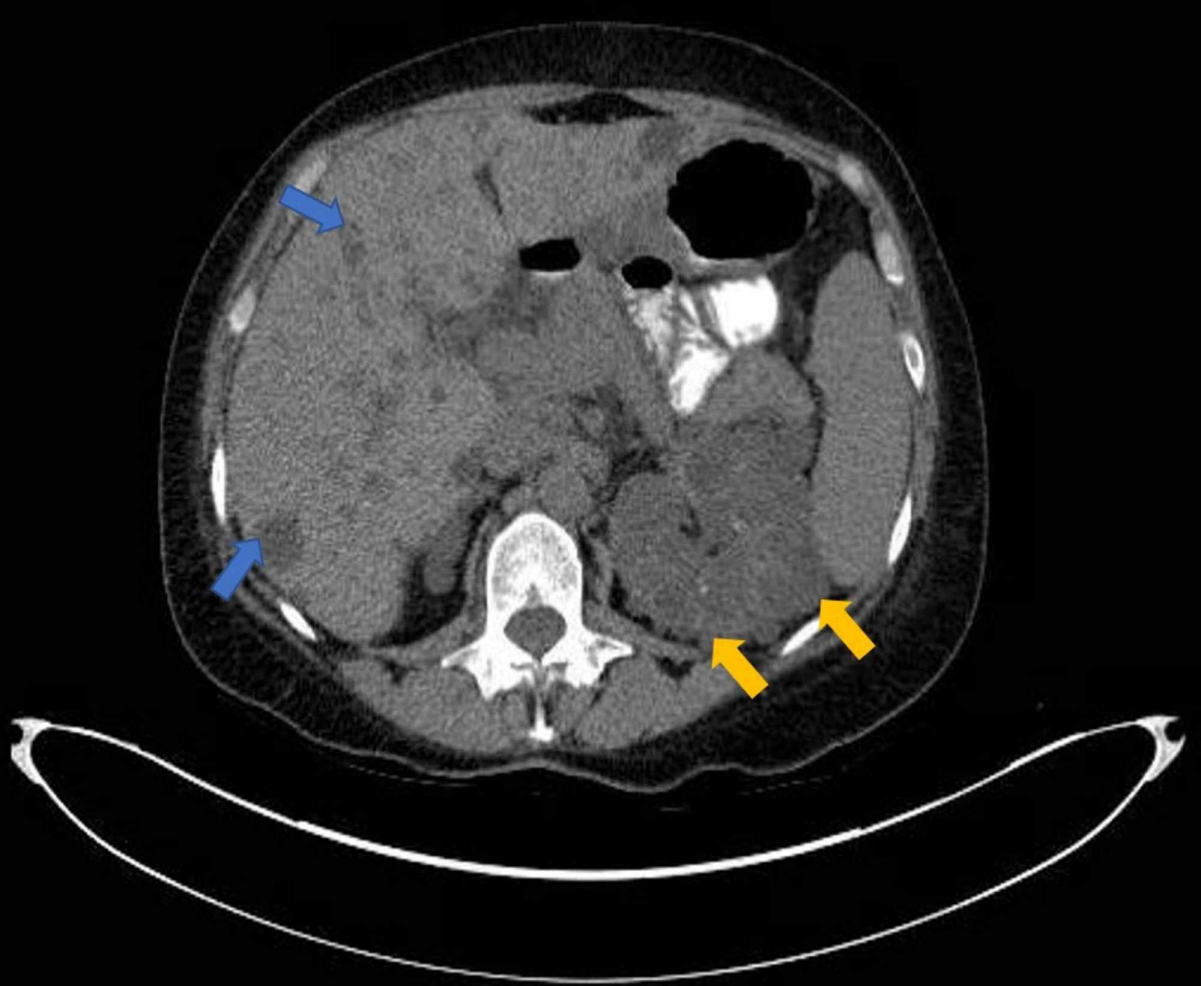
Computed tomography (CT) scan of abdomen and pelvis showing presence of cysts in liver (blue arrows) and cysts in the kidney (yellow arrows)

**Figure 2:**
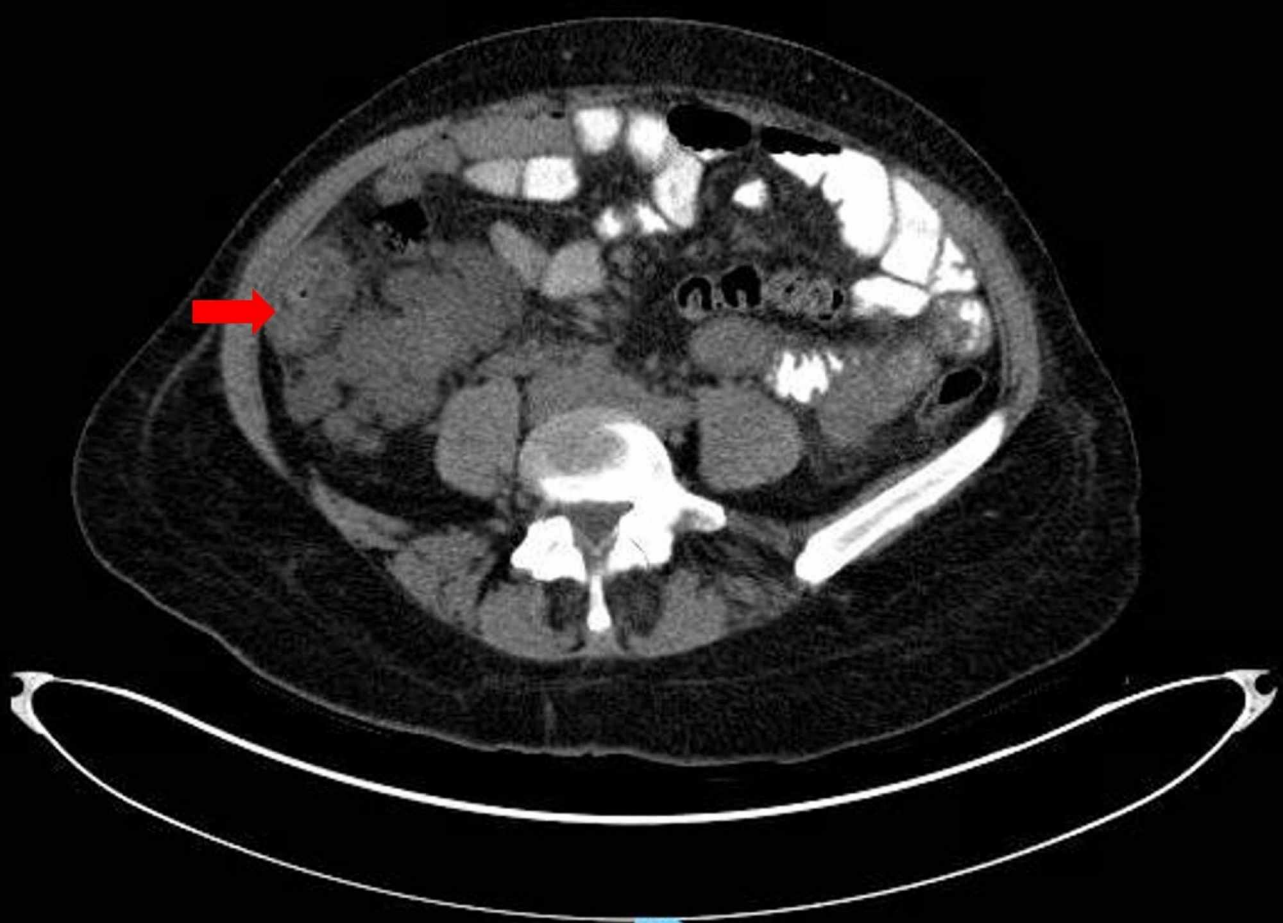
Computed tomography (CT) scan of abdomen and pelvis showing thickening of wall of ascending colon with mild inflammatory changes (red arrow), suggestive of colitis of the ascending colon

Admission labs were significant for white blood cell (WBC) 2,070/mm^3^ [normal range (N): 3,800-10,500/mm^3^], absolute neutrophil count (ANC) 1,865/mm^3^ [N: 1,800-7,400/mm^3^], lymphocyte count 500/mm^3^ [N: 1,000-3,300/mm^3^], platelets 108,000/mm^3^ [N: 150,000-400,00/mm^3^], aspartate transaminase (AST) 81 U/l [N: 10-40 U/l], alanine transaminase (ALT) 87 U/l [N: 10-45 U/l], alkaline phosphatase (ALP) 137 U/l [N: 40-120 U/l] and normal renal function. Electrocardiogram revealed sinus rhythm with no acute changes. Chest radiography and computed tomography (CT) ruled out the presence of pneumonia. Abdominal ultrasound showed polycystic liver and kidneys, contracted gallbladder with negative sonographic Murphy’s sign. CT of the abdomen and pelvis confirmed polycystic liver and kidneys, as well as mural thickening of ascending colon with mild surrounding inflammatory changes (suggestive of colitis) (Figures 1, 2).

Urinalysis was not suggestive of infection. Respiratory viral panel multiplex test was negative. Clostridium difficile toxin test was negative. Stool cultures were negative. Stool microscopy was negative for ova and parasites. Urine cultures revealed Escherichia coli (10,000-49,000 CFU/ml) combined with normal urogenital flora. Blood cultures revealed no growth. Testing for acute hepatitis A, B and C viruses was negative. Epstein-Barr virus serology revealed prior infection. BK virus DNA, Parvovirus B19 DNA and adenovirus DNA were not detected by specific polymerase chain reaction (PCR) tests.

Infectious diseases service was consulted. Given the presence of leukopenia, thrombocytopenia, transaminitis and multi-systemic symptoms, there was concern for an ongoing viral process. She was started on valganciclovir 900 mg PO BID for presumed CMV disease, with intravenous fluid support. The E. coli in the urine was thought to be a colonization and she was successfully monitored off antibiotics.

She continued to have persistent fevers and diarrhea, with worsening leukopenia (lowest WBC count 1700/mm^3^ with lowest ANC 800/mm^3^), thrombocytopenia (nadir 86,000/mm^3^) and liver function abnormalities (AST 139 U/l, ALT 135 U/l, ALP 167 U/l). Given the persistent symptoms and worsening laboratory parameters, MMF was discontinued. Serum CMV PCR testing revealed 154,697 IU/ml (5.19 log10 IU/ml) [lower limit of assay detection 60 IU/ml (1.78 log10 IU/ml)]. Gastroenterology service was consulted for worsening liver function abnormalities and they attributed the clinical picture to systemic CMV infection.

She had clinical improvement, with improving CMV PCR tests [504 IU/ml (2.70 log10 IU/ml)] after 12 days of antiviral therapy. She was discharged on the twelfth day of hospital stay, with recovery of WBC count to 5,600/mm^3^ and platelets 147,000/mm^3^.

She was followed up by in the outpatient transplant nephrology and infectious diseases clinics. CMV PCR was negative 60 days after initiation of antiviral therapy and she was restarted on a lower dose of MMF. After the second negative CMV PCR a week later, MMF dose was increased and valganciclovir was decreased to prophylactic dosing. She received valganciclovir for a total of 87 days.

Patient 2

A 62-year-old female with past medical history of endometriosis, hypertension, chronic kidney disease (likely from anti-inflammatory drug use for management of endometriosis) was being followed in the transplant nephrology clinic. She received a deceased donor kidney transplant in 1998 that failed in March 2018 and was subsequently on hemodialysis. She received a second renal transplant from a deceased unrelated donor in June 2019.

She received the Influenza vaccine at the end of September 2019. On routine outpatient labs performed a week later, she was noted to have detectable CMV viral levels [4,983 IU/ml (3.70 log10 IU/ml)] without any symptoms. The levels continued to rise over the course of two months [214,823 IU/ml (5.33 log10 IU/ml)], with no improvement after switching from valganciclovir 450 mg QD to valganciclovir 900 mg QD. She was noted to have worsening leukopenia (WBC 1,260/mm^3^), so she was admitted for further management.

On admission, she reported multiple episodes of non-bloody, foul-smelling diarrhea. She denied sick contacts, recent travel, medication changes or medication non-compliance. Her home medications consisted of tacrolimus ER 8 mg QD, prednisone 5 mg QD, MMF 1000 mg BID, sulfamethoxazole-trimethoprim single-strength tablet QOD, valganciclovir 450 mg QD.

She was hemodynamically stable on examination. Physical examination revealed no abnormalities. Admission labs revealed WBC count 1,480/mm^3^ (ANC 950/mm^3^ and lymphocyte count 270/mm^3^), hemoglobin 11.1 g/dl (N: 13-17 g/dL), blood urea nitrogen (BUN) 20 mg/dL [N: 7-23 mg/dL], creatinine 1.53 mg/dl [N: 0.50-1.30 mg/dL], ALP 124 U/l. Chest radiograph revealed clear lungs. BK virus and Parvovirus B19 DNA PCR tests were negative.

MMF and valganciclovir was held on admission. Infectious diseases service was consulted and she was started on renally-dosed intravenous (IV) ganciclovir (2.5 mg/kg QD) and letermovir 480 mg QD PO. Given the lack of response to outpatient valganciclovir therapy, a sample was sent for CMV resistance testing. With improvement in her creatinine clearance, the ganciclovir dosing was subsequently adjusted. WBC nadir during the hospital stay was 1,450/mm^3^ (ANC 780/mm^3^), with subsequent recovery due to intravenous antivirals and one dose of filgrastim.

CMV resistance tests revealed the presence of two different populations of virus - wild type and a strain exhibiting resistance at M460V and C603W (two out of seven mutations conferring resistance to ganciclovir). No resistance was noted to cidofovir or foscarnet. She was discharged on IV ganciclovir and oral letermovir. The final duration of therapy would be determined by outpatient follow-up with infectious diseases and transplant nephrology. She was to continue tacrolimus ER 3 mg QD PO and prednisone 5 mg QD PO at home.

## Discussion

There are multiple risk factors linked to CMV viremia and disease in SOTRs and after hematopoietic stem cell transplant. These include older age, female sex, blood group A, decreased creatinine clearance, allograft impairment, CMV serostatus prior to transplant (donor seropositive-recipient seronegative OR recipient seropositive), transplantation type, use of T-cell depleted stem cell grafts, use of anti-thymocyte globulin before transplant, and occurrence of graft-versus-host disease (GvHD) [2-6]. Vaccination has not been previously reported as a risk factor for CMV viremia.

The influenza virus vaccine administered seasonally is a trivalent or quadrivalent inactivated vaccine [1]. It is a T cell-dependent common recall antigen and has been shown to induce T cell activation and type 1 cytokine production (such as interleukin-2 and interferon-γ) [7]. There is a paucity of data on the effects of influenza vaccination leading to viremia in solid organ transplant recipients. Thus, we reviewed the data available in human immunodeficiency virus (HIV)-infected patients, another subset of immunocompromised patients.

The research on the effect of influenza virus on HIV replication has produced conflicting results. Some studies have shown that influenza vaccination results in increased HIV replication (and subsequently increased viremia) in patients with uncontrolled or partially controlled viral replication [7-10]. Other studies have shown that vaccination did not result in increased HIV replication [11-16]. It is believed that a transient increase in plasma HIV RNA levels occurs during the first and second weeks after vaccination - this may have been missed in some studies due to less frequent sampling [13]. A randomized controlled trial comparing the effect of vaccines (versus placebo) on HIV transcription and immune activation reported an increased HIV transcription, without increase in HIV DNA or plasma HIV RNA [17]. Vaccination is seen to produce a systemic inflammatory response - this may cause upregulation of viral production from chronically infected cells or activation of latently infected bystander cells [8,18]. This ultimately leads to an increase in RNA levels in patients who have ongoing low-level viral replication [8].

In some transplant recipients, influenza-like illness has been reported after vaccination [19]. SOTRs are maintained in a state of prolonged immunosuppression after transplant. Thus, there is a possibility that influenza vaccination may lead to transient increase in CMV viral load and subsequent viremia (similar to the transient viremia seen in HIV patients). The exact mechanisms leading to viremia are not known - however the potential role of estrogen in transduction of CMV genes [2], as well as the effects of systemic inflammatory response (induced by vaccination) on viral replication [8,18] indicate that molecular and genetic mechanisms may play a role.

Both patients had a temporal association of onset of CMV viremia with administration of the influenza vaccine (Table 1). They were older females at high risk of CMV reactivation (donor CMV seropositive/recipient CMV seronegative). Patient 2 was on CMV prophylaxis, while patient 2 was not receiving valganciclovir (per our institutional protocol of CMV prophylaxis until six months post-transplant). They reported compliance with their home medications, with no recent increases in their maintenance immunosuppression. Patient 1 had an episode of antibody-mediated rejection, while patient 2 had an upper respiratory bacterial infection in the preceding months - these resolved completely with appropriate therapy. They had no concurrent inflammatory disease processes at the time of vaccination. Lab testing demonstrated high CMV DNA levels with concomitant leukopenia, neutropenia and lymphopenia. There were no additional factors identified to have contributed to CMV viremia. The CMV viremia was severe enough to warrant therapeutic antiviral therapy and changes in their immunosuppression regimen. We thus believe that CMV viremia resulted from the prior vaccination in our patients.

**Table 1:** Patient characteristics ALT: Alanine transaminase; ANC: Absolute neutrophil count; ALP: Alkaline phosphatase; AST: Aspartate transaminase; BID: Every 12 hours; BUN: Blood urea nitrogen; CMV: Cytomegalovirus; ER: Extended release; IV: Intravenous; IVIG: Intravenous immune-globulin; PO: Oral; QD: Every twenty-four hours; QID: Every six hours; QOD: Every forty-eight hours; SMX-TMP: Sulfamethoxazole-trimethoprim; SS: Single-strength; WBC: White blood cell count.

Sr. No.	Characteristic	Patient 1	Patient 2
1	Type of donor	Living unrelated donor	Deceased donor
2	Initial transplant / Re-transplant	Initial transplant	Re-transplant
3	Donor CMV status	CMV seropositive	CMV seropositive
4	Recipient CMV status	CMV seronegative	CMV seronegative
5	Induction immunosuppressive regimen	1. Anti-thymocyte globulin 2. Methylprednisolone 3. IVIG	1. Anti-thymocyte globulin 2. Methylprednisolone
6	Recent inflammatory disease processes (allograft rejection, infection)	Donor-specific antibody mediated rejection	Upper respiratory infection
7	Time period between transplant and inflammatory disease process	4 months	1 month
8	Treatments for inflammatory disease processes	1. IVIG 2. Rituximab 3. Apheresis	Amoxicillin
9	Duration of inflammatory disease process	1 month	1 week
10	Prior episodes of CMV reactivation	None	None
11	Primary / Secondary CMV prophylaxis	Off CMV prophylaxis at the time of disease presentation	Primary prophylaxis
12	Recent increase in maintenance immunosuppressive regimen	None	None
13	Age at time of presentation	55 years	62 years
14	Time period between transplant and presentation	17 months	3 months
15	Time period between vaccination and presentation	8 days	7 days
16	Prophylactic medications at the time of presentation	SMX-TMP SS 1 tablet PO QD	SMX-TMP SS 1 tablet PO QOD Valganciclovir 450 mg QD
17	Immunosuppressive regimen at the time of presentation	Prednisone 5 mg PO QD MMF 360 mg PO QID Tacrolimus 0.5 mg PO BID	Prednisone 5 mg QD MMF 1000 mg BID Tacrolimus ER 8 mg QD
18	Initial symptoms	Fevers, chills, night sweats lethargy, headaches, myalgias, nausea, anorexia, pleuritic chest pain watery, non-bloody diarrhea	Watery, non-bloody diarrhea
19	Initial laboratory abnormalities	WBC 2,070/mm^3^ ANC 1,865/mm^3^ Platelets 108,000/mm^3^ AST 81 IU/l ALT 87I U/l ALP 137 IU/l	WBC 1,480/mm^3^ ANC 950/mm^3^ Hemoglobin 11.1 g/dl BUN 20 mg/dl Creatinine 1.53 mg/dl ALP 124 IU/l
20	Initial lymphocyte count [N: 1,000-3,300/mm^3^]	500/mm^3^	270/mm^3^
21	CMV viral load [lower limit of assay detection: 60 IU/ml (1.78 log10 IU/ml)]	154,697 IU/ml (5.19 log10 IU/ml)	214,823 IU/ml (5.33 log10 IU/ml)
22	Treatment regimen	Valganciclovir 900 mg PO BID	1. Ganciclovir 2.5 mg/kg IV QD (initial dosing) 2. Letermovir 480 mg PO QD
23	Changes in immunosuppressive regimen	Temporary discontinuation of MMF	1. Temporary discontinuation of MMF 2. Decrease in Tacrolimus dosing (Tacrolimus ER 3 mg QD PO)
24	Patient outcome	Control of CMV viremia and recovery of cell counts	Control of CMV viremia and recovery of cell counts
25	Graft outcome	Stable allograft function	Stable allograft function

One meta-analysis concluded that the influenza vaccine was associated with statistically significant protection against influenza-like illness in immunocompromised patients, including SOTRs [20]. Given the significant proven benefits of vaccination in SOTRs (versus the risk of potential viremia), we firmly believe that all SOTRs should get the inactivated influenza vaccine and other vaccines as per the latest guidelines [1]. Through this case report, we wish to highlight the finding of CMV viremia occurring after influenza vaccination, with no other identified triggers. Further research and accumulation of cases is needed to investigate the strength of this association, to determine the mechanisms that resulted in CMV viremia and to recognize the risk factors leading to progression from viremia to clinically relevant CMV disease.

## Conclusions

Vaccination may lead to transient viral replication in immunocompromised patients, such as HIV-infected patients (likely due to genetic and immune-mediated mechanisms). Similarly, we believe that influenza vaccination led to transient CMV viremia and CMV disease in our renal transplant recipients on potent immunosuppressive therapies. Further research is needed to assess the strength of this correlation, the mechanisms causing viremia and the risk of progression to clinical disease. Until then, clinicians must continue vaccination of SOTRs per the guidelines, but must also remember to enquire about recent vaccination when evaluating SOTRs with new onset CMV viremia.
